# Creation of fertility-restored materials for Ogura CMS in *Brassica oleracea* by introducing *Rfo* gene from *Brassica napus* via an allotriploid strategy

**DOI:** 10.1007/s00122-020-03635-8

**Published:** 2020-07-01

**Authors:** Hai-long Yu, Zhi-yuan Li, Wen-jing Ren, Feng-qing Han, Li-mei Yang, Mu Zhuang, Hong-hao Lv, Yu-mei Liu, Zhi-yuan Fang, Yang-yong Zhang

**Affiliations:** Institute of Vegetables and Flowers, Chinese Academy of Agricultural Sciences, No. 12 ZhongGuanCun South St., Beijing, 100081 People’s Republic of China

## Abstract

**Key message:**

Ogura CMS fertility-restored materials, with 18 chromosomes, normal seed setting, stable fertility and closer genetic background to the parent Chinese kale, were successfully developed in *B. oleracea* via a triploid strategy for the first time.

**Abstract:**

Ogura cytoplasmic male sterility (CMS) is the most widely used sterile type in seed production for commercial hybrids of *Brassica oleracea* vegetables. However, the natural Ogura CMS restorer line has not been found in *B. oleracea* crops. In this study, the triploid strategy was used with the aim to create euploid *B. oleracea* progenies with the *Rfo* gene. The allotriploid AAC hybrid YL2 was used as a male parent to backcross with Ogura CMS Chinese kale. After successive backcrosses, the BC_2_ *Rfo*-positive individual 16CMSF2-11 and its BC_3_ progenies, with 18 chromosomes, were developed, which were morphologically identical to the parent Chinese kale. Compared with F_1_ and BC_1_ plants, it showed stable fertility performance, and regular meiosis behavior and could produce seeds normally under natural pollination. The genomic composition analysis of *Rfo*-positive progenies by using molecular markers showed that more than 87% of the C-genome components of BC_3_ *Rfo*-progenies recovered to the parent Chinese kale, while most or all of the *A*_*n*_-genome segments were lost in 16CMSF2-11 and its progenies. The results suggested that the genetic background of *Rfo*-positive individuals was closer to that of the parent Chinese kale along with backcrossing. Hereof, the Ogura CMS fertility-restored materials of Chinese kale were successfully created via triploid strategy for the first time, providing a bridge for utilizing the Ogura CMS *B. oleracea* germplasm in the future. Moreover, our study indicates that the triploid strategy is effective for transferring genes from *B. napus* into *B. oleracea*.

**Electronic supplementary material:**

The online version of this article (10.1007/s00122-020-03635-8) contains supplementary material, which is available to authorized users.

## Introduction

*Brassica oleracea* is a typical cross-pollination species, and its F_1_ hybrids exhibit strong heterosis, such as the high yield, better quality, increased uniformity, wide adaptability and resistance to biotic stresses in single cross-hybrids of cabbage, cauliflower and broccoli (Fang et al. 1983; Pearson 1983; Kucera et al. 2006; Singh et al. 2009, 2019; Dey et al. 2014). Thus, development of F_1_ hybrids is one of the most important objectives for *B. oleracea* vegetables. Due to the size and structure of *B. oleracea* flowers, it is not cost-effective to produce commercial hybrid seeds by manual emasculation and pollination. Instead, the male-sterile (MS) breeding system is a widely used, effective method in the production of commercial hybrids of *B. oleracea* vegetables (Fang et al. 2004; Yamagishi and Bhat 2014).

Genic male sterility (GMS) and cytoplasmic male sterility (CMS) are two main types of male sterility systems used in hybrid seed production (Kumar et al. 2000; Chen and Liu 2014). Most natural GMS mutants are difficult to utilize in the hybrid seed production due to their recessive traits (Nieuwhof 1961; Dickson 1970; Fang et al. 2001). In CMS system, the naturally occurring CMS resources are absent in *B. oleracea*, and most of the CMS types used in *B. oleracea* were transferred from radish, *Brassica napus* and other related cruciferous species through distant hybridization (Thompson 1972; Pearson 1972; Yarrow et al. 1990; Bannerot et al. 1974; Shu et al. 2016). Ogura CMS is a spontaneous mutational CMS type discovered in a natural population of radish, which is stable in sterility and easy to transfer (Ogura 1968). Owing to its excellent sterile characteristics, through efforts over several generations, Ogura CMS has been successfully transferred to *B. oleracea* by distant hybridization and protoplast fusion (Bannerot et al. 1974; Walters et al. 1992; Dey et al. 2011). Currently, Ogura CMS is the most widely used CMS type in hybrid seed production for *B. oleracea* vegetables (Wang et al. 2012; Dey et al. 2013; Singh et al. 2019).

However, due to all the Ogura CMS germplasm could not be self-pollinated, the excellent Ogura CMS germplasm cannot be utilized. For instance, clubroot disease is now becoming an increasingly severe disease, and resistant germplasm resources are absent in *B. oleracea* vegetables. A few clubroot-resistant varieties of cabbage are available on the market; for example, Tuoni and XG336 show high resistance to clubroot disease. However, they cannot be reutilized due to their Ogura CMS cytoplasm (Zhang et al. 2016; Ning et al. 2018). The development of *B. oleracea* Ogura CMS restorer lines is of great importance for the innovation and utilization of Ogura CMS germplasm. However, to date, a natural Ogura CMS restorer line has not been found in *B. oleracea* crops.

Previously, to obtain Ogura CMS fertility-restored lines in *B. oleracea*, the restorer-fertility gene (*Rfo*) for Ogura CMS was successfully transferred from rapeseed into Chinese kale by interspecific hybridization combined with embryo rescue. Fertility-restored interspecific hybrids (YL2, ACC) have been obtained (Yu et al. 2016). Due to the poor fertility of allotriploid ACC, colchicine doubling was performed to improve the fertility of the interspecific hybrids. The interspecific hexaploid hybrids (YL2-3, AACCCC) were produced and used as the male parent for backcrossing with Ogura CMS Chinese kale to develop BC generations (Yu et al. 2017). However, fertility-restored BC progenies with different genetic backgrounds still exhibited polyploid compositions, abnormal meiotic behaviors and showed a low seed setting rate (less than one seed per pod) under natural pollination (Yu et al. 2018).

In the present study, to speed up the process of creating Ogura CMS restorer materials in *B. oleracea*, the allotriploid AAC hybrid YL2 was used as the male parent to backcross with Ogura CMS Chinese kale, with the expectation of isolating euploid C gametes derived from the *Rfo*-positive progenies without chromosome doubling. With marker-assisted selection (MAS), morphology, fertility, cytological observations and genetic component analysis, Ogura CMS fertility-restored materials of Chinese kale (2*n* = 18) were developed.

## Materials and methods

### Plant materials

The F_1_ interspecific allotriploid fertility-restored individual (code: YL2, ACC), with an average pollen viability of 36.5%, was developed by distant hybridization between Chinese kale and rapeseed in a previous study (Yu et al. 2016). In this study, the F_1_ triploid individual YL2 was used as the male parent to produce BC_1_ plants. A high-generation Ogura CMS Chinese kale (code: 15Y102) was chosen as the recurrent female parent to develop BC generations. All of the plants used in the present study were grown in a greenhouse in autumn under normal management.

### Development of BC progenies and screening of *Rfo*-positive individuals

The F_1_ fertility-restored individual YL2 was backcrossed with Chinese kale 15Y102 by repeated hand pollination during the bud period. Due to the interspecific reproductive barrier and low seed setting rate, embryo rescue was performed after hand pollination in the BC_1_ generations. Immature pods at 10 days after pollination were removed from plants and cultured in vitro, and then, mature embryos were excised from these pods after culturing for 10–15 days. The details of embryo rescue were described by Yu et al. (2016).

**Fig. 1:**
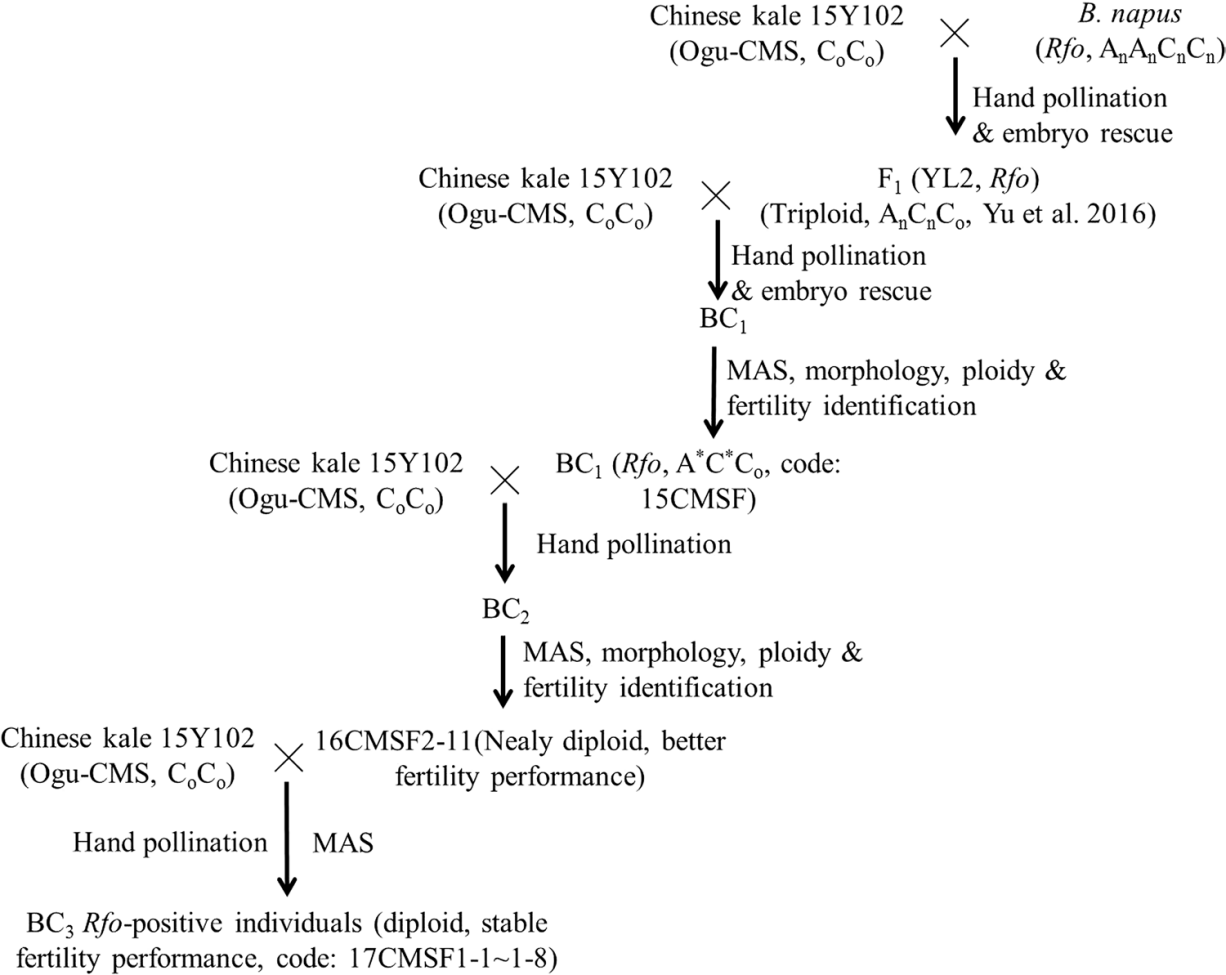
A diagram of breeding process for creating Ogura CMS fertility-restored materials via triploid strategy in Chinese kale

The *Rfo*-positive individuals in the BC_1_ and BC_2_ generations, showing relatively good fertility performance, were chosen as pollen donors to backcross with the parent 15Y102 for developing the next generations by hand pollination (without embryo rescue). The backcross procedure is shown in Fig. 1.

During the seed harvest period, the number of pollinated pods and seeds harvested from each cross-combination were investigated. The seed setting rate (seeds per pod) was calculated. After growing to seedlings, the individuals harboring the *Rfo* gene were screened for the *Rfo*-specific marker (Bn*RFO*-AS2F/Bn*RFO*-NEW-R) (Yu et al. 2016). All *Rfo*-positive individuals were propagated in plant regeneration medium (MS medium with 1 mg l^−1^ 6-BA and 0.1 mg l^−1^ NAA) to ensure pollen supply. The rooted seedlings were transferred into soil pots and cultivated in a greenhouse.

### Morphological traits and fertility performance investigation

The morphological characteristics, including the plant type, color of basal leaves, shape of basal leaves, margin of basal leaves, leaf surface of basal leaves, wax on leaf surfaces, flower color and inflorescence type of the *Rfo*-positive individuals, were investigated according to the standards described in “Descriptors and data standards for Chinese kale” (Sun and Li 2008).

At anthesis, the fertility performance of the *Rfo*-positive individuals was assessed by measuring pollen viability using the acetocarmine dyeing method. The pollen grains were collected from three newly opened flowers and stained with 1% acetocarmine. More than 300 pollen grains were observed in each replication. Pollen viability was observed once every 10 days. The percentage of viable pollen, which was stained deep pink and had a plump shape, was calculated from three replications.

### Ploidy identification and cytological analysis

The ploidy of *Rfo*-positive progenies was identified through flow cytometry (FCM, BD FACSCalibur™, BD Biosciences, San Jose, CA, USA). The nuclear DNA content was estimated according to the method of Dolezel et al. (2007) with some modifications. The specific procedure was as follows: (1) a small amount (approximately 200 mg) of plant leaf tissue was placed in a petri dish; (2) 1–1.5 ml of ice-cold Galbraith’s buffer for nucleus isolation was added, and the tissue was cut immediately in the buffer with a sharp scalpel; (3) the homogenate was mixed and then filtered through a 37-mm nylon mesh into a 1.5-ml sample tube (approximately 0.5 ml of filtrate); (4) the supernatant was carefully removed, and PI stock solution (50 mg ml^−1^ with 50 mg ml^−1^ RNase) was added; (5) the sample was incubated on ice (15–30 min) with occasional shaking before FCM analysis. Then, the Chinese kale parental DNA content (2C) was measured as per the reference. The G1 peak was positioned on the abscissa (200 channels) by adjusting the gain settings of the instrument. The coefficient of variation (CV) of all samples was below 5%.

For meiotic analysis, young floral buds were collected and treated with 8-hydroxyquinoline for 3–4 h at room temperature. Then, the samples were rinsed with distilled water and fixed in Carnoy’s solution [alcohol:acetic acid, 3: 1 (vol.)] for 24 h at room temperature and stored in 70% ethanol at 4 °C (Li et al. 2014). Anthers were separated and hydrolyzed by an enzyme mix (0.2% pectinase and 0.2% cellulase) for 4–5 h at 37 °C and then squashed and stained with PI solution (50 μg ml^−1^). Chromosome pairing at diakinesis and chromosome segregation at anaphase were observed and analyzed for 50 pollen mother cells (PMCs) in each BC *Rfo*-positive plant.

### Indel marker design for genomic component analysis and polymorphism detection

To identify the background markers between the parents, resequencing of the parents at 30 × coverage over the whole genome (Chinese kale 15Y102 and *B. napus* 15Y403) was performed. This work was completed at the Beijing Genomics Institute (BGI) (Shenzhen, China). The resequencing data of the two parents were mapped to the TO1000 reference genome of *B. oleracea* (*C*_*o*_ genome, http://plants.ensembl.org/Brassica_oleracea) using BWA (http://biobwa.sourceforge.net/index.shtml, Li and Durbin 2009) and then sorted by SAMtools (http://samtools.sourceforge.net). The 3–8 bp insertion-deletion mutations (Indels) between the parents were called using the Genome Analysis Toolkit (GATK), with the filter criteria of a QUAL value larger than 30 and a minimum depth of 10. Then, 150-bp flanking sequences on both sides of these Indels were extracted and searched against the *B. napus* reference genome (http://www.genoscope.cns.fr/brassicanapus/). Only unique and perfectly matched sequences located on the *C*_*n*_ genome were retained to avoid multiple loci on the *B. napus* reference genome. Primers were designed along chromosomes with intervals of 20 kb and with amplicon lengths varying from 100 to 200 bp, primer lengths ranging from 18 to 23 bp, GC contents of 40–50% and *T*_*m*_ values of 52–56 °C.

Because the *A*_*n*_ genome was specific to *B. napus*, specific *A*_*n*_-markers covering the whole *A*_*n*_ genome were developed as follows: First, the *A*_*n*_-genome sequences acquired from the *B. napus* reference genome (http://www.genoscope.cns.fr/brassicanapus/) were segmented into 500-bp fragments and marked with location information; then, these 500-bp sequences were aligned to the *C*_*o*_ reference genome (TO1000) using BWA-MEM. A length of 6,432,500 bp of the *A*_*n*_-genome-specific sequences, which could not be aligned to the *C*_*o*_ reference genome (TO1000) genome, was retained and used to design primers by Primer3. The design principles were the same as those mentioned above.

In this study, two or three pairs of primers were selected at each interval of 5 Mb in the whole *C*_*o*_ and *A*_*n*_ reference genomes, respectively. Finally, a total of 300 pairs of *C*_*o*_-genome primers and 156 pairs of *A*_*n*_-genome primers were selected to detect polymorphisms between the parents (Supplementary Table 1).

**Table 1:** Comparison of the seed set and proportion of *Rfo*-positive individuals among the BC_1_, BC_2_, and BC_3_ generations

Generation	Number of pods	Number of seeds (embryo excised)	Seed setting rate (seeds/pod)	Number of viable plants	Number of *Rfo*-positive individuals	*Rfo*-positive individual code
BC_1_ (without embryo rescue)	3173	14	0.004 ± 0.0003^a^	5	1	15CMSF-Y1
BC_1_ (with embryo rescue)	234	342		3	0	
BC_2_	2022	217	0.111 ± 0.0105^b^	167	2	16CMSF2-11, 16CMSF2-58
BC_3_	3282	18,533	5.605 ± 0.3750^c^	8736	8	17CMSF1-1 ~ 17CMSF1-8

Values followed by the different superscript letters indicate have significant difference at *P *= 0.01, based on the least significant difference test

### DNA extraction and genetic background marker detection

Genomic DNA was extracted from fresh leaves using a modified cetyltrimethylammonium bromide (CTAB) protocol (Murray and Thompson 1980). The concentration of DNA was estimated using a spectrophotometer (BioDrop, UK) and adjusted to 40–50 ng/μL.

Polymerase chain reaction (PCR) experiments were performed in 10 µL reaction mixture containing 2 µL DNA template, 1 µL 10 × PCR buffer (Mg^2+^ included), 0.8 µL dNTPs (2.5 mM each), 0.4 µL forward and reverse primers (10 µM), 0.1 µL Taq DNA polymerase (5 U/µL), and 5.3 µL double-distilled H_2_O. PCR was performed using the following program: 94 °C for 5 min; 35 cycles of 94 °C for 30 s, 55 °C for 30 s, and 72 °C for 45 s; and 72 °C for 10 min. The amplicons were separated by 8% (w/v) polyacrylamide gel electrophoresis (160 V for 1.5 h) and visualized with silver nitrate staining (Bassam et al. 1991).

## Results

### Development of BC progenies and *Rfo*-positive screening

**Fig. 2:**
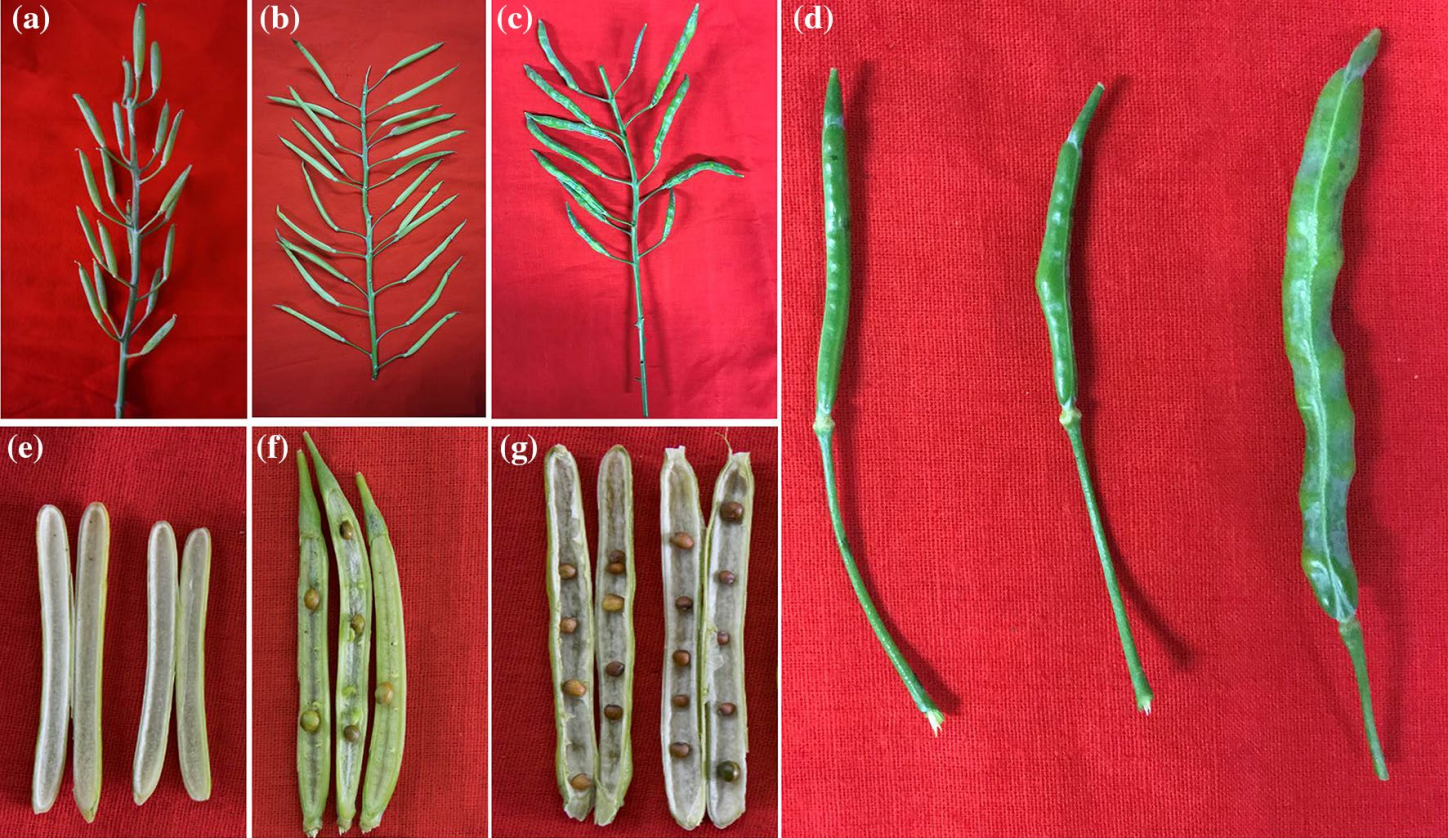
The seed setting performance of recurrent parent 15Y102 when using YL2 (F_1_), 15CMSF-Y1 (BC_1_), and 16CMSF2-11 as pollen donors, respectively. **a**–**c** Inflorescences morphology of 15Y102 in 30 DAPs (days after pollination) after pollinated with YL2 (**a**), 15CMSF-Y1 (**b**), and 16CMSF2-11 (**c**), respectively. **d** Comparison of the pod morphology of 15Y102 in 40 DAPs after pollinated with YL2, 15CMSF-Y1 and 16CMSF2-11 (from left to right). **e**–**g** Comparison of seed growth in pods of 15Y102 in 50 DAPs after pollinated with YL2 (**e**), 15CMSF-Y1 (**f**) and 16CMSF2-11 (**g**), respectively

Successive backcrossing and MAS of *Rfo* were performed in the BC_1_–BC_3_ generations (Fig. 1, Table 1). The seed set of *Rfo*-positive individuals was investigated and compared among BC_1_–BC_3_ *Rfo*-positive individuals. Most of the pollinated pods with pollen of YL2 failed to elongate and expand, and had no seeds inside (Fig. 2a, e, d). Only 14 seeds survived without embryo rescue, with an average of 0.004 seeds per pod (14/3173) (Table 1). When using embryo rescue, 342 mature embryos were excised from 234 pods (1.47 embryos per pod on average). Many embryos excised from pods were collapsed, malformed or shriveled. Only three seedlings were obtained (Table 1). Finally, a total of eight BC_1_ seedlings were obtained through hand pollination and embryo rescue. Upon screening with the *Rfo*-specific marker, only one BC_1_ individual (code: 15CMSF-Y1), harboring the *Rfo* gene, was identified (Table 1).

Then the individual 15CMSF-Y1, as the male parent, was backcrossed with the Ogura CMS Chinese kale 15Y102 to produce the BC_2_ progenies. A total of 6094 flower buds were pollinated, and 217 seeds were harvested from 2022 pods (Table 1). Similar to YL2, most of the harvested pods pollinated with the pollen of 15CMSF-Y1 had no seeds (Fig. 2b, d, f). Compared with the F_1_ individual YL2, the average seed setting rate of 15CMSF-Y1 was 0.11 seeds per pod, showing significant (*P *< 0.01) improvement. These seeds were sown, and 167 of the seeds germinated and grew into seedlings. Upon screening with the *Rfo*-specific marker, two *Rfo*-positive individuals, named 16CMSF2-11 and 16CMSF2-58, were detected.

The individual 16CMSF2-11, showing a better fertility performance in the flowering stage, was selected as a pollen donor for development of the BC_3_ generation. Unlike the F_1_ individual (YL2) and BC_1_ individual (15CMSF-Y1), the pollinated pods of 16CMSF2-11 expanded obviously with 6–10 plump seeds per pod. Relatively, few seeds were aborted during pod development (Fig. 2c, d, g). The seed setting rate of 16CMSF2-11 exhibited a normal level (average of 5.7 seeds per pod), which was significantly higher than those of F_1_ (YL2) and BC_1_ (15CMSF-Y1) (*P *< 0.01), and had no significant difference from that of the maintainer line of 15Y102 (*P *< 0.05). In the BC_3_ generation, a total of 18,533 seeds were harvested, and 10,000 seeds were randomly selected to sow for MAS screening (Table 1). A total of 8736 BC_3_ progenies were grown. Upon screening with the *Rfo*-specific marker, eight out of the 8736 BC_3_ progenies were positive (code: 17CMSF1-1 ~ 17CMSF1-8).

### Morphological characterization and fertility performance of *Rfo*-positive individuals

**Fig. 3:**
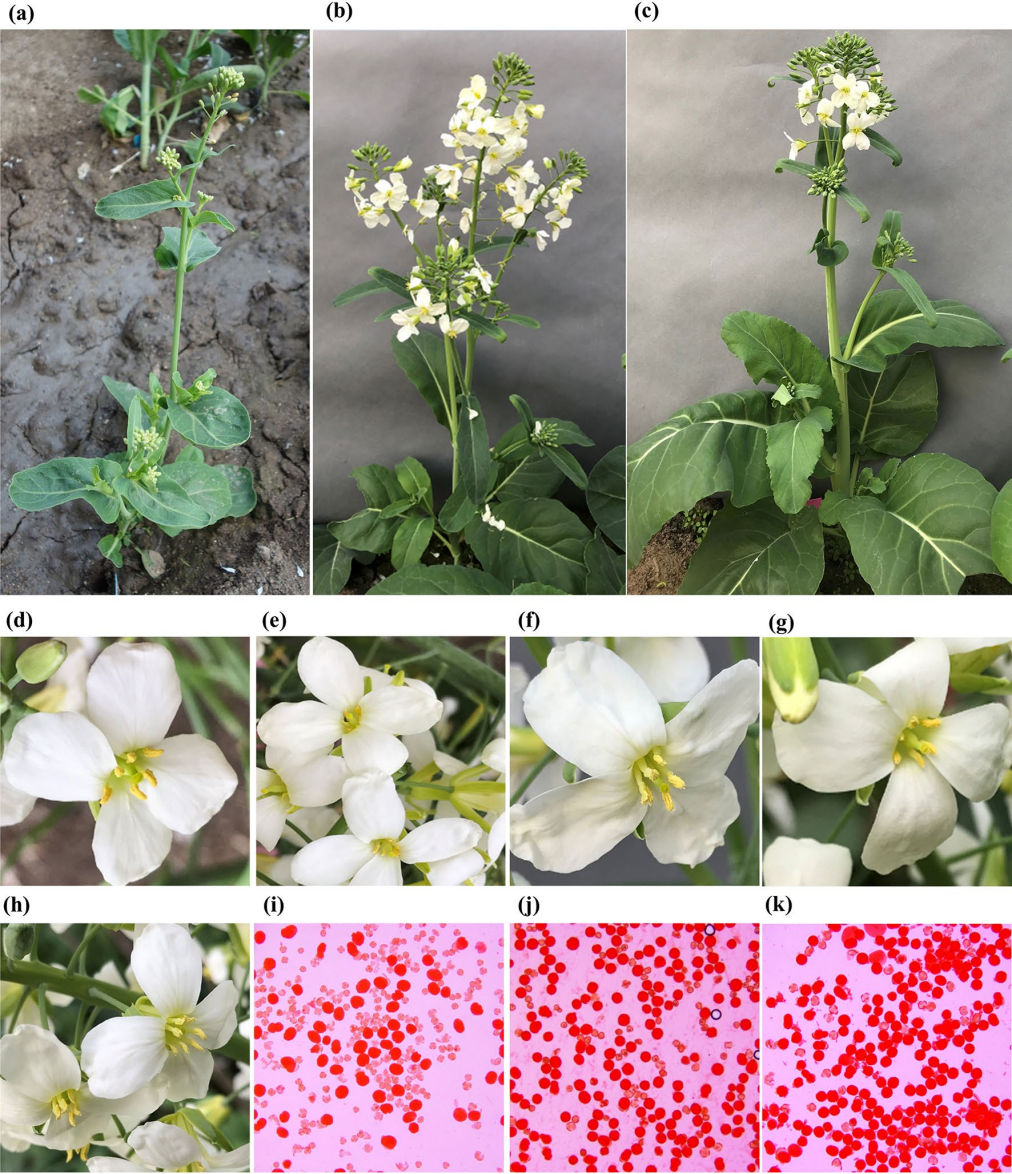
Morphological characterization (**a**–**c**) and fertility performance (**d**–**k**) of the BC_1_ hybrid 15CMSF-Y1, BC_2_ hybrid 16CMSF2-11 and BC_3_ hybrid 17CMSF1-1, respectively. Plant morphology of 15CMSF-Y1 (**a**), 16CMSF2-11 (**b**) and 17CMSF1-1 (**c**). **d, e** Pollen performance of 15CMSF-Y1 during different flowering periods; **f**–**h** Pollen performance of 16CMSF2-11 (**f**), 16CMSF2-58 (**g**) and 17CMSF1-1 (**h**); **i**–**k** Pollen viability of 15CMSF-Y1 (**i**), 16CMSF2-11 (**j**) and 17CMSF1-1 (**k**)

Morphological characteristics, including leaf, bud, inflorescence, and flower, of *Rfo*-positive BC progenies were investigated. Unlike the interspecific F_1_ hybrid YL2, which has an intermediate morphology between Chinese kale and rapeseed (Yu et al. 2016), the BC_1_ individual 15CMSF-Y1 was morphologically similar to the parent Chinese kale 15Y102 (Fig. 3a). For example, the plant type (semi-erect), plant height (52.45–64.32), leaf shape (elliptical), leaf color (gray green with wax deposition) and flower color (nearly white) were all similar to those of Chinese kale (Fig. 3a, d), but there remained some differences from Chinese kale, such as thinner plant type and abnormal inflorescence (Fig. 3a). Compared with the BC_1_ individual 15CMSF-Y1, the morphology-related traits, including the plant type, leaves shape and flower color (entirely white) of two BC_2_ and eight BC_3_ *Rfo*-positive individuals were identical to those of the parent Chinese kale (Fig. 3b, c). The inflorescences of 10 BC_2_ and BC_3_ *Rfo*-positive individuals grew normally and had no abnormal or dead buds during plant growth (Fig. 3). Moreover, the BC_2_ and BC_3_ *Rfo*-positive individuals grew more vigorously than the parent Chinese kale 15Y102, and their flowering stage occurred was 10 to 15 days earlier than that of 15Y102.

At anthesis, one BC_1_ (15CMSF-Y1), two BC_2_ (16CMSF2-11 and 16CMSF2-58) and eight BC_3_ *Rfo*-positive individuals were fertility-restored (Fig. 3d–k). For the BC_1_ *Rfo*-positive individual 15CMSF-Y1, the fertility performance was unstable during the whole flowering period (Fig. 3d, e). Its pollen viability varied throughout the flowering period, ranging from 15% to 65%, with the mean pollen viability of 45.5% (Fig. 3i). At the late flowering stage (after the 30th day of flowering), most flowers of 15CMSF-Y1 could not produce pollen grains (Fig. 3e). Compared with the BC_1_ individual 15CMSF-Y1, the BC_2_ individual 16CMSF2-11 showed a better and more stable fertility performance (Fig. 3f). The pollen viability of 16CMSF2-11 was always above 65%, with an average pollen viability of 76.2% (Fig. 3j), showing significant improvement compared with that of 15CMSF-Y1. Furthermore, all eight BC_3_ individuals showed stable fertility performance throughout the flowering period (Fig. 3h), and their mean pollen viability was all above 85% (Fig. 3k).

### Ploidy identification and meiotic behaviors of *Rfo*-positive individuals

**Fig. 4:**
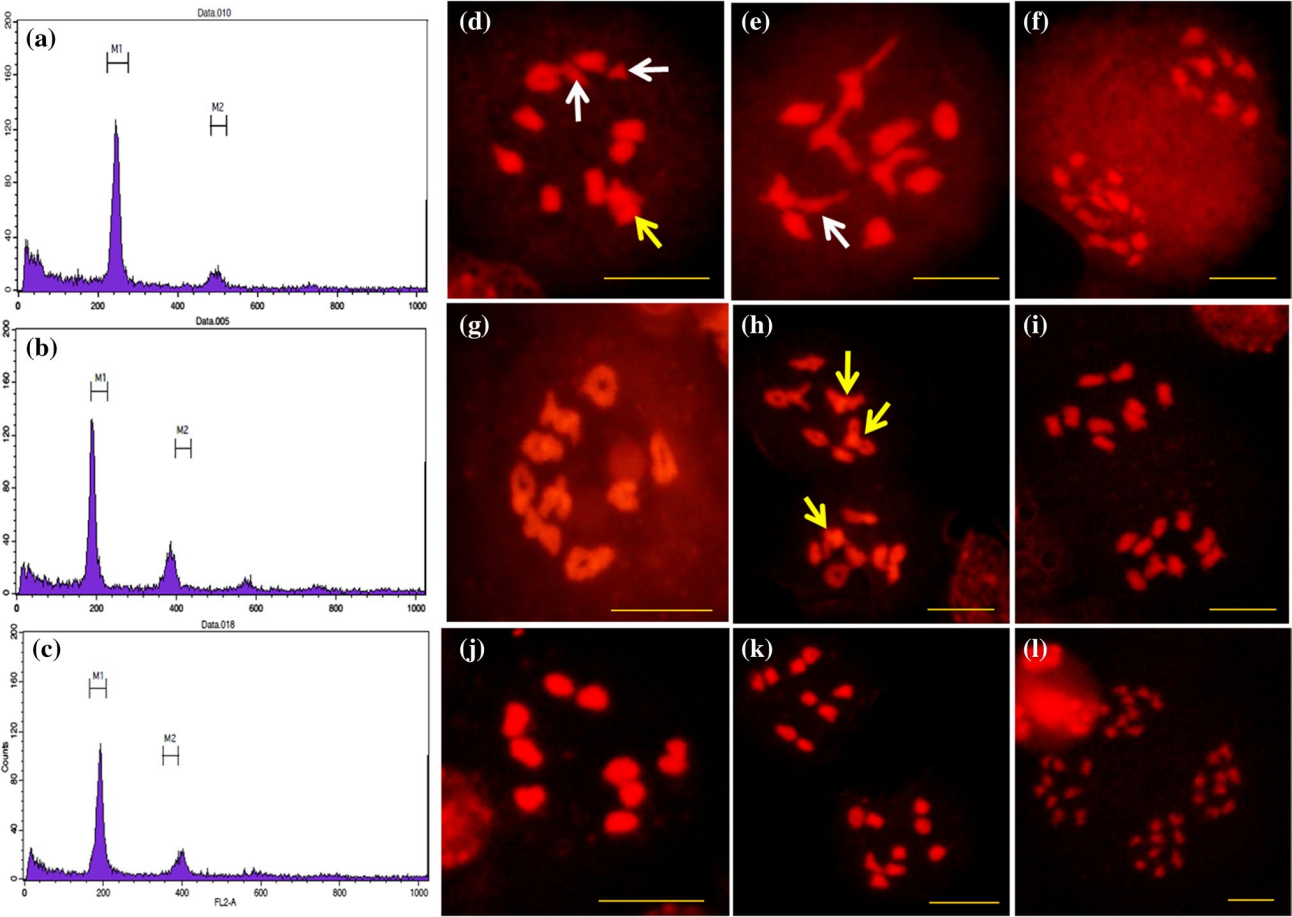
Ploidy identification (**a**–**c**) and cytogenetic characterization (**d**–**l**) of the BC_1_ hybrid 15CMSF-Y1, BC_2_ hybrid 16CMSF2-11 and BC_3_ hybrid 17CMSF1-1. Ploidy identification (relative nuclear DNA content) of 15CMSF-Y1 (**a**), 16CMSF2-11 (**b**) and 17CMSF1-1 (**c**) by FCM. **d, e** The PMC of 15CMSF-Y1 at diakinesis with abnormal chromosome configuration (**d, e**); multivalents (yellow arrows) and univalents (white arrows) were frequently observed. **f** One PMC of 15CMSF-Y1 with segregation of 9:13. **g, h** One PMC of 16CMSF2-11 at diakinesis with chromosome configuration 9II (**g**); multivalents (yellow arrows) and univalents were observed (**h**). **i** One PMC of 16CMSF2-11 with segregation of 9:9. **j** One PMC of 17CMSF1-1 at diakinesis with chromosome configuration 9II. **k** One PMC of 17CMSF1-1 equally segregated at anaphase I with chromosome segregation of 9: 9. **l** One PMC of 17CMSF1-1 equally segregated at anaphase II formed tetrad. Scale bars = 10 µm (color figure online)

To determine the ploidy level, the DNA content of *Rfo*-positive individuals was estimated using flow cytometry. The G1 peak positions of 15CMSF-Y1 (BC_1_), 16CMSF2-11 (BC_2_) and 16CMSF2-58 (BC_2_) were located in 244 channels (Fig. 4a), 195 (Fig. 4b) and 205 channels, respectively, which suggested that their ploidy was close to that of the parent Chinese kale. The ploidy levels of all BC_3_ *Rfo*-positive plants, whose G1 peak positions located in 200 channels (Fig. 4c), were consistent with the ploidy of Chinese kale, suggesting that all eight BC_3_ *Rfo*-positive plants had returned to normal diploid level.

The cytological analysis revealed that multivalents and univalents were observed in all PMCs of the BC_1_ *Rfo*-positive individual 15CMSF-Y1 at diakinesis, with the number of bivalents ranging from 6 to 10 (Fig. 4d, e). Moreover, all 50 PMCs produced unequal chromosome separation at anaphase I, with high frequency of the 10:12 and 9:13 chromosome distribution patterns (Fig. 4f). The chromosome number of most PMCs in 15CMSF-Y1 was 22 at anaphase I (Fig. 4f), yet some PMCs showed less than 22 chromosomes, which suggested that some chromosomes may be lost during the meiosis process. With regard to the two BC_2_ *Rfo*-positive individuals, the chromosome number of 16CMSF2-11 was 18 (Fig. 4i), while that of 16CMSF2-58 was 19. Abnormal chromosome behavior, including multivalents, univalents, and abnormal chromosome pairings could still be found in most PMCs of 16CMSF2-11 at diakinesis (Fig. 4h). However, the normal chromosome configuration of 9II was found in 6% of the PMCs of 16CMSF2-11 (3/50, Fig. 4g). In addition, 24% (12/50) of the PMCs showed a chromosome distribution pattern of 9:9 at anaphase I (Fig. 4i), indicating the possibility of producing euploid progenies with 18 chromosomes.

**Fig. 5:**
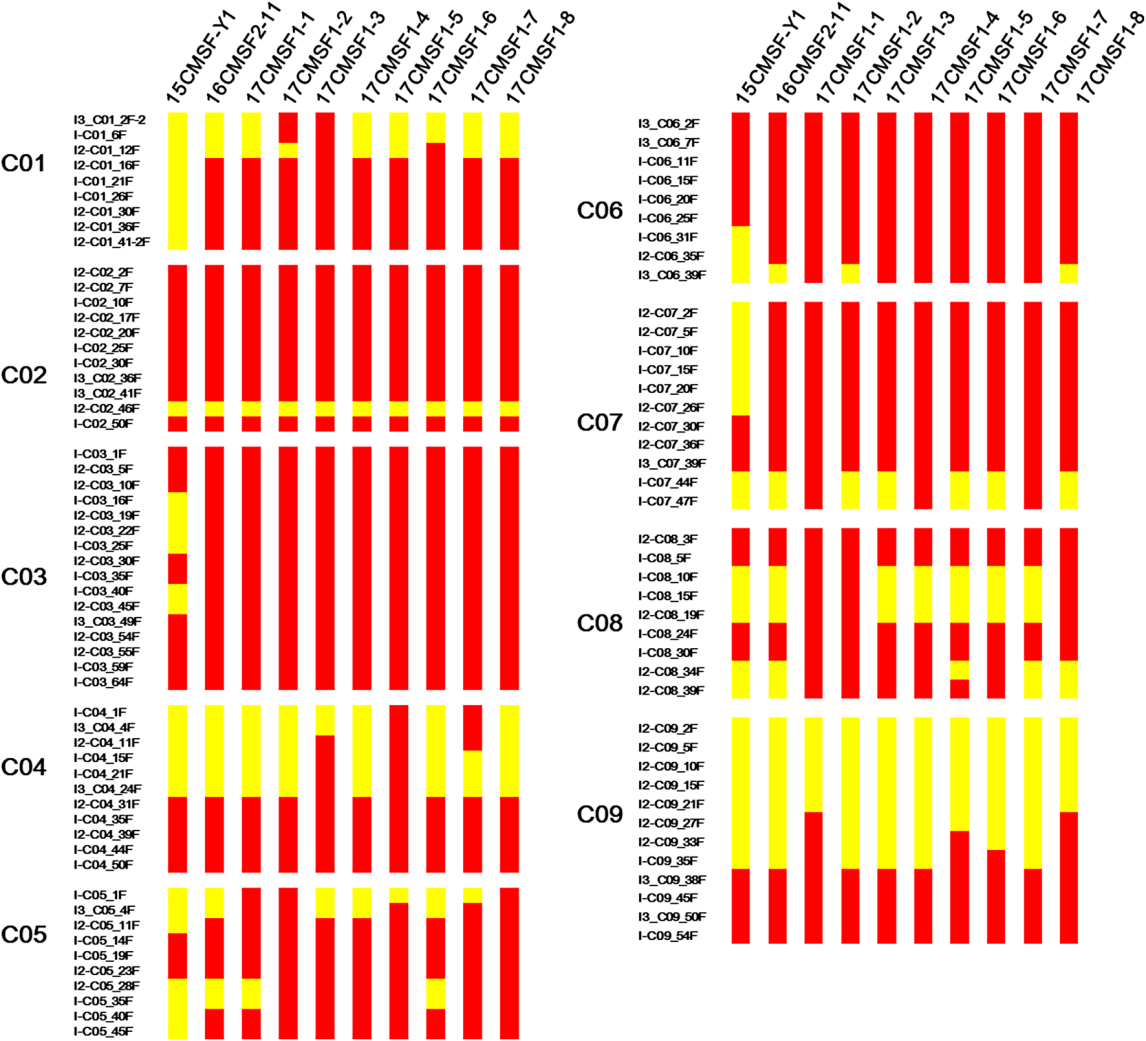
Analysis of the genomic composition of the BC_1_ hybrid 15CMSF-Y1, BC_2_ hybrids 16Q2-11, and BC_3_ hybrids 17CMSF1-1–17CMSF1-8 by the *C*_*o*_-genome primers. The marker name and marker locations are listed to the left of each chromosome. Red: loci of parent Chinese kale 15Y102 types. Yellow: loci of heterozygous types

All eight BC_3_ individuals displayed a similar meiotic behavior at diakinesis, and the chromosomes pairing become more normal (Fig. 5j). All eight BC_3_ individuals had 18 chromosomes identical to 16CMSF2-11. The most frequently occurring configuration of 18 chromosomes, 9II, was observed in more than 55% of the analyzed PMCs (Fig. 5j), and the proportions were 64% (32/50), 70% (35/50), 72% (36/50), 56% (28/50), 84% (42/50), 60% (30/50), 60% (30/50) and 76% (38/50) for 17CMSF1-1 to 17CMSF1-8, respectively.

### Genomic composition analysis of *Rfo*-positive individuals

A total of 98 pairs of *C*_*o*_-genome polymorphic primers between parents were used to analyze the genomic components of the BC_1_–BC_3_ *Rfo*-positive individuals (Supplementary Fig. S1). In 15CMSF-Y1, 45 out of 98 pairs of *C*_*o*_-genome primers amplified identical single band patterns as those of the parent Chinese kale 15Y102 (indicated with red in Fig. 5), while the remaining 53 primers (indicated with yellow in Fig. 5) showed heterozygous bands. The results showed that 72.96% of the 15CMSF-Y1 genomic components of *C*_*o*_-genome had recovered to that of the parent 15Y102. For the two BC_2_ *Rfo*-positive individuals (16CMSF2-11 and 16CMSF2-58), 68 and 66 pairs of *C*_*o*_-genome primers amplified identical bands as the parent 15Y102, which suggested that 83.67% and 84.69% of the *C*_*o*_-genome components, respectively, had been recovered to those in the parent 15Y102 (Fig. 5). The genomic components of the *C*_*o*_ genome in the eight BC_3_ *Rfo*-positive individuals were closer to those of the parent Chinese kale, and more than 87% (87.24–91.32%) of the *C*_*o*_-genome components were the same as those in the parent Chinese kale (Fig. 5). The individuals 17CMSF1-1 and 17CMSF1-5 contained the highest proportion of *C*_*o*_-genome components, while 17CMSF1-6 had the lowest recovery proportion. Genomic composition analysis of eight BC_3_ *Rfo*-positive individuals indicated that chromosome C09 had the highest proportion of heterozygosity compared with the other eight chromosomes (Fig. 5).

**Fig. 6:**
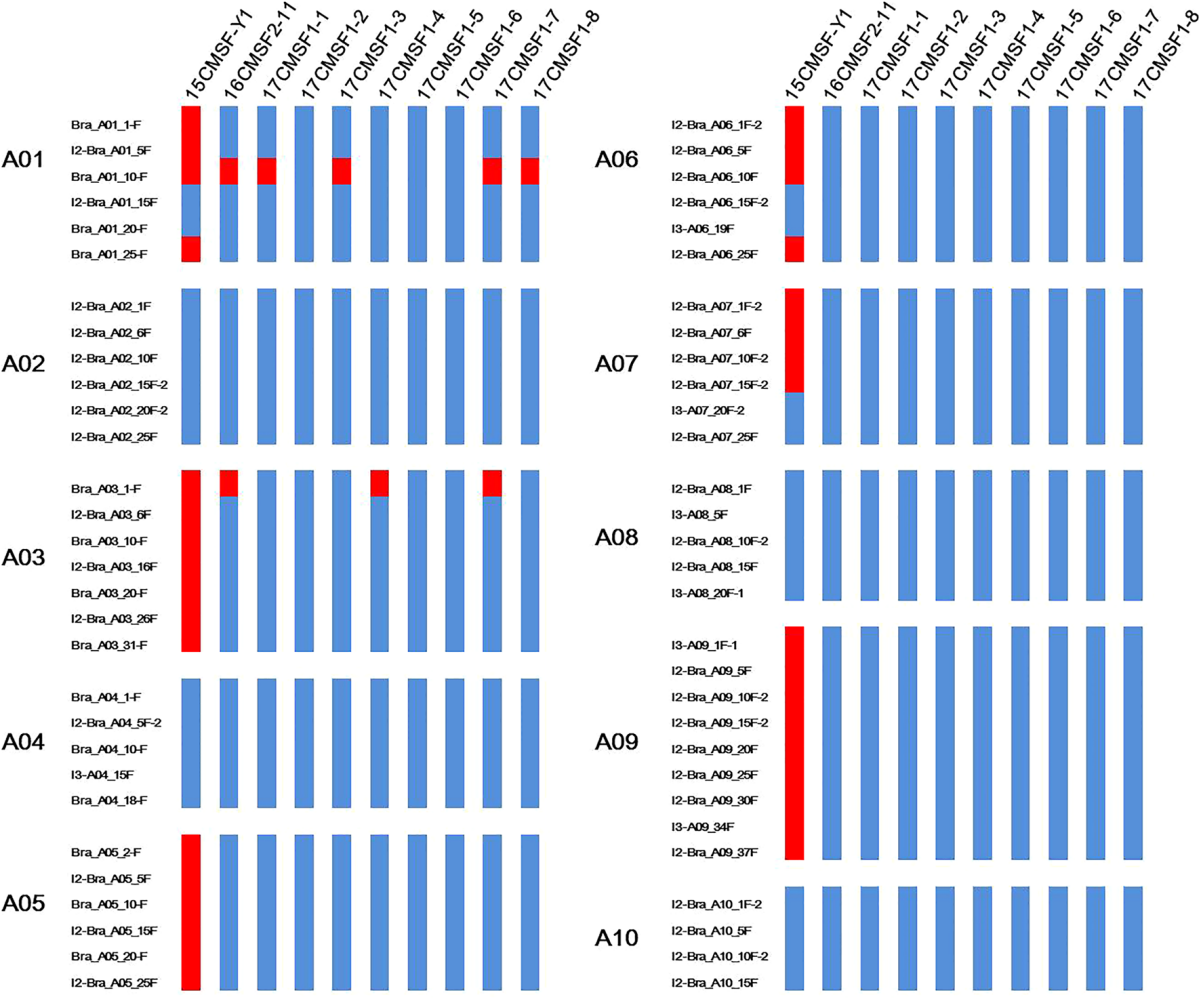
Analysis of the genomic composition of the BC_1_ hybrid 15CMSF-Y1, BC_2_ hybrid 16Q2-11 and BC_3_ hybrids 17CMSF1-1–17CMSF1-8 by the *A*_*n*_-genome specific primers. The marker name and marker locations are listed to the left of each chromosome. Red: presence of parent *B. napus* 15Y403 fragments. Blue: absence of parent *B. napus* 15Y403 fragments

For the *A*_*n*_ genome, only 60 pairs of primers could amplify single clear bands in the parent rapeseed 15Y403 and F_1_ individual YL2 but not in Chinese kale 15Y102, and these primers were used to analyze the genomic component of the *A*_*n*_-genome in the BC_1_–BC_3_ *Rfo*-positive individuals (Fig. 6). Among the 60 pairs of *A*_*n*_-genome primers, 26 pairs were absent in the BC_1_ *Rfo*-positive individual 15CMSF-Y1 (indicated with blue in Fig. 6), which indicated that these loci were lost in 15CMSF-Y1. For the two BC_2_ *Rfo*-positive individuals, only 2 pairs and 5 pairs of primers were present in 16CMSF2-11 and 16CMSF2-58 (Fig. 6), respectively. In the BC_3_ generation, these two loci were lost in 17CMSF1-2, 17CMSF1-5 and 17CMSF1-6, which suggested that the *A*_*n*_-genome was completely eliminated in these three BC_3_ *Rfo*-positive individuals (Fig. 6), whereas the other five BC_3_ *Rfo*-positive individuals retained a small number of *A*_*n*_-genome fragments derived from the parent *B. napus* (Fig. 6).

## Discussion

Ogura CMS is the most widely exploited cytoplasm in the F_1_ hybrid production of *Brassica* vegetables throughout the world (Sakai and Imamura 1990; Dey et al. 2011; Yamagishi and Bhat 2014). It plays an important role in exploiting *Brassica* vegetable heterosis and improving agricultural yield (Fang et al. 2004; Dey et al. 2017). Furthermore, the Ogura CMS system provided excellent protection for F_1_ commercial hybrids at the technical level because all of the offspring exhibited MS, especially in *B. oleracea* vegetables that lacked the natural Ogura CMS restorer line. However, on the other hand, the creation of restorer line might challenge the protection of varieties with this cytoplasm, because some breeders only used Ogura CMS to protect the varieties instead of the plant variety protection (PVP) system. The concept of essentially derived varieties (EDVs), developed in UPOV (1991), specified that the EDV may qualify for protection if authorization is obtained from the breeder of the initial variety (IV), aiming to provide better protection to the breeder of the IV. However, the concept is not equivalent to denying the rights of breeders of EDVs, and it encourages all forms of plant breeding, thereby also increasing the motivation for germplasm innovation (Jördens 2005). Taking knowledge from some crops with natural restoration materials, such as rice and peppers (Shifriss and Frankel 1971; Kumar et al. 2007; Chen and Liu 2014; Kim and Zhang 2018), breeders should rely mainly on PVP to protect their varieties. For the protected Ogura CMS varieties, we would like to encourage that the breeder to use the fertility-recovered Ogura CMS germplasm to develop IV, or EDVs with the authorization of the breeder of the IV.

In this work, the Ogura CMS fertility-restored materials in *B. oleracea* were successfully created for the first time. The Ogura CMS fertility-restored materials could provide a bridge for germplasm reutilization of Ogura CMS. For example, clubroot-resistant cabbage germplasms are very scarce in *B. oleracea*, and a few Ogura clubroot-resistant varieties of cabbage are available on the market (Zhang et al. 2016). Recently, we successfully transferred a widely used clubroot resistance gene from Ogura clubroot-resistant materials to our inbred line with a normal cytoplasm by bridge material 16CMSF2-11, which lays a solid foundation for improving clubroot resistance in varieties of *B. oleracea* (Ren et al. 2020). Thus, the development of the fertility-restored Ogura CMS material provides a bridge for the utilization of the Ogura CMS materials in *B. oleracea*.

Distant hybridization was widely used as an important tool for increasing genetic variation, gene introgression and cultivated crop improvement in plants (Kalloo 1992; Liu et al. 2014). Alien chromosome lines (substitution, addition and translocation), obtaining in the process of distant hybridization, play an important role in transferring a beneficial trait from wild plants into crops, especially in wheat (Qi et al. 2007; Yang et al. 2009; Jiang et al. 1994; Mei et al. 2015). Previously, chromosomal doubling was performed, and the amphidiploid individual (AACCCC) was obtained (Yu et al. 2016, 2017, 2018). Amphidiploids are usually used as a bridge for the development of alien gene introgression or alien chromosome lines (Liu et al. 2014). However, the genomic introgression of rapeseed was difficult to eliminate by continuous backcrossing, and the diploid progenies were difficult to obtain, possibly because of homoeologous chromosome pairing between *B. napus* and *B. oleracea* (Song et al. 1995; Xiong et al. 2011; Xiong & Pires 2011). As an alternative strategy, in our study, the Ogura CMS restorer gene (*Rfo*) was successfully introduced into *B. oleracea* from *B. napus* via an allotriploid strategy. The results indicated that pollen viability (45.5%) was very poor in the BC_1_ hybrids, which resulted in a low seed setting rate (11.1%), and very few BC_2_ seeds (217) were produced (Table 1). This result is also consistent with a previous study on interspecific crossing between *B. oleracea* and *B. napus* (Chiang et al. 1977; Ayotte et al. 1987; Quazi 1988; Inomata 2002; Ripley and Beversdorf 2003; Li et al. 2014). Due to reproductive isolation between parents, the cross-compatibility barrier, complex character separation and poor fertility of hybrid offspring are usually observed in the process of distant hybridization (Comai 2000; Kumar et al. 2011). Cytological analysis results also suggested that abnormal chromosomal behaviors occurred very frequently in the BC_1_ generations (Fig. 4). As backcrossing continued, there was a dramatic increase in fertility (76.2%) and seed set (6–10 seeds per pod) in the BC_2_ individual 16CMSF2-11 and its progenies (Fig. 3). Furthermore, their chromosome behaviors became more normal during meiosis (Fig. 4). Combined with the genomic composition results (Figs. 5, 6), we inferred that alien chromosome lines (substitution or translocation) containing the *Rfo* gene were successfully developed, and introgression of *C*_*n*_ components from *B. napus* to *B. oleracea* was shown to be available via the allotriploid strategy between the two genomes in our study. Thus, our study provides a reference strategy for transferring genes into *B. oleracea* from *B. napus*.

In the process of distant hybridization, it is necessary to confirm the genomic composition and chromosome recombination of the progenies. Cytological identification, in situ hybridization (ISH) and molecular markers are the most common methods used to analyze genomic composition and meiotic behavior in distant hybrids (Kerlan et al. 1993; Li et al. 1995; Snowdon et al. 1997; Snowdon 2007; Heneen et al. 2012; Wen et al. 2012; Younis et al. 2015; Yu et al. 2015). However, due to the small genome size and high homology between the A/C genome and A/C subgenome, cytological identification and ISH have many limitations in application (Xiong and Pires 2011). Molecular markers are also widely used to identify the genomic composition of *Brassica* interspecific hybrids (Navabi et al. 2011; Fredua-Agyeman et al. 2014). In this study, Indel markers, evenly distributed throughout the genome, were used to analyze the genomic composition of *Rfo*-positive individuals of the BC_1_–BC_3_ generations. With this approach, we found that except at Chr. C01, recombination occurred at the C chromosomes in 15CMSF-Y1, and the homozygous (*C*_*o*_/*C*_*o*_) blocks recovered nearly 50% of the C genome in the BC_1_ hybrid (Fig. 5). With successive backcrossing with Chinese kale, a large number of genomic components of the *C*_*o*_-genome had recovered to those in the parent 15Y102 (Fig. 5). Interestingly, the proportions of *C*_*o*_-genome blocks in BC_2_ (83.67% and 84.69%) and BC_3_ (87.24–91.32%) *Rfo*-positive individuals were lower than the expected value (87.5%, 93.75%). Two main possible reasons for this result can be proposed: (1) the introgression fragment linked with the *Rfo* gene, such as the genomic composition of chromosome C09, has a tendency to be heterozygous for the *C*_*n*_/*C*_*o*_ genome (Fig. 6), as observed in the process of developing a *B. napus* fertility-restored line (Delourme et al. 1998). (2) Conservation of more *C*_*n*_ genome regions from *B. napus* is necessary in *Rfo*-positive individuals for plant survival, similar to the study of Pele et al. (2017). Additionally, for the *A*_*n*_ genome, more than 50% of the loci in A chromosomes were lost in the BC_1_ hybrid 15CMSF-Y1 and most locus in some A chromosomes was completely eliminated in 16CMSF2-11 and its BC_3_ progenies (Fig. 6). Combined with the chromosome number of 15CMSF-Y1, we inferred that the alien chromosomes A03, A05 and A09 were additional chromosomes in the 15CMSF-Y1. Some chromosomes were partially eliminated in 15CMSF-Y1, most likely because of homoeologous exchanges between the A and C genomes (Ding et al. 2015; Yang et al. 2017; Pele et al. 2017; Li et al. 2018). Furthermore, the introgression of *A*_*n*_ fragments from *A*_*n*_ chromosomes was eliminated in BC_3_ hybrids, and even completely eliminated in some BC_3_ hybrids (Fig. 6). In summary, a small proportion of *C*_*n*_ genomic components from *B. napus* was present in *Rfo*-positive individuals, and the genomic composition of *Rfo*-positive individuals was complex, as shown by molecular markers, which probably explains why the transmission rate of *Rfo* was abnormal. Therefore, further backcross is needed to eliminate the *B. napus* chromosome fragments under the condition of maintaining the *Rfo* gene in future.

## Electronic supplementary material

Below is the link to the electronic supplementary material.

Supplementary Fig. S1. Screening results for the *C*_*o*_-genome primers (a) and *A*_*n*_-genome primers (b) between male parent 15Y403 and female parent 15Y102. The black box indicates that those primers show polymorphismbetween the parents 15Y102 and 15Y403 or amplify single clear bands in 15Y403 (JPEG 48 kb)

Supplementary material 2 (XLSX 48 kb)
