# Supplementary figures and images for: Creation of fertility-restored materials for Ogura CMS in Brassica oleracea by introducing Rfo gene from Brassica napus via an allotriploid strategy
Source: Theor Appl Genet. 2020 Jul 1;133(10):2825–37. doi: 10.1007/s00122-020-03635-8 (PMC7497352; doi:10.1007/s00122-020-03635-8)

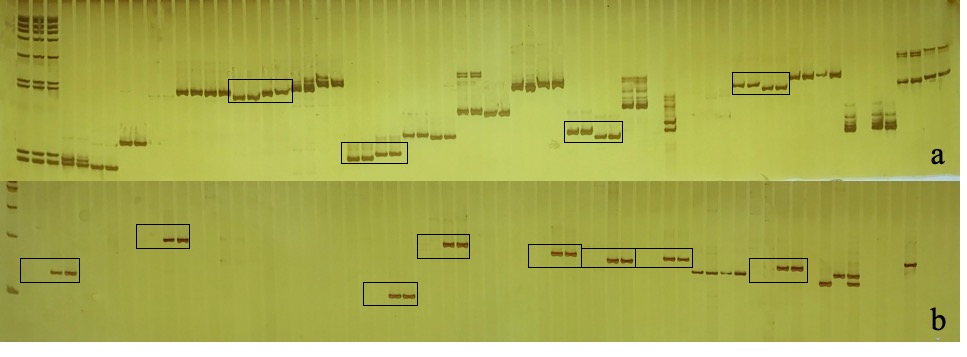

Supplement: Supplementary file 1 — Supplementary Fig. S1. Screening results for the Co-genome primers (a) and An-genome primers (b) between male parent 15Y403 and female parent 15Y102. The black box indicates that those primers show polymorphismbetween the parents 15Y102 and 15Y403 or amplify single clear bands in 15Y403 (JPEG 48 kb) [file 122_2020_3635_MOESM1_ESM.jpg]
